# Integrated Cardiovascular–Kidney–Metabolic Disease Burden in Mexico: Trends from 2000 to 2023 and Projections to 2050

**DOI:** 10.5334/aogh.5558

**Published:** 2026-11-10

**Authors:** Rubén Silva-Tinoco, María Fernanda Bernal-Ceballos, Alejandro Avalos-Bracho, Lilia Castillo-Martínez, Vladimir Martínez-De la Cruz, Paloma Almeda-Valdes, José Manuel Arreola-Guerra, Daniel Sierra Lara-Martínez

**Affiliations:** 1 Servicios Públicos de Salud del Instituto Mexicano del Seguro Social para el Bienestar, Mexico City, Mexico; 2 Centro Nacional de Prevención y Control de Enfermedades, Secretaría de Salud, Mexico City, Mexico; 3 Departamento de Nutrición, Instituto Nacional de Ciencias Médicas y Nutrición Salvador Zubirán, Mexico City, Mexico; 4 Departamento de Endocrinología y Metabolismo, Unidad de Investigación de Enfermedades Metabólicas, Instituto Nacional de Ciencias Médicas y Nutrición Salvador Zubirán, Mexico City, Mexico; 5 Instituto de Atención Integral de Enfermedades Renales del Estado de Aguascalientes, Aguascalientes, Mexico; 6 Unidad de Cuidados Críticos Coronarios, Instituto Nacional de Cardiología Ignacio Chávez, Mexico City, Mexico

**Keywords:** cardiovascular-kidney-metabolic health, disease burden, disability-adjusted life-years, Mexico, global burden of disease, forecasting

## Abstract

*Background:* Cardiovascular–kidney–metabolic (CKM)-related diseases are interconnected, yet their collective burden in Mexico remains unquantified.

*Objective(s):* To quantify the magnitude, composition, temporal trends, geographic divergence, and projected trajectory of CKM-related disease burden in Mexico.

*Methods:* We analyzed Global Burden of Disease 2023 estimates of CKM-related burden among Mexican adults aged 20 years or older; standard all-age age-standardized rates were used for comparisons with Latin America and the Caribbean and globally. Mutually exclusive causes were grouped into cardiovascular, kidney, type 2 diabetes, and metabolic dysfunction-associated steatotic liver disease domains. We quantified disability-adjusted life-years (DALYs) during 2000–2023 and used 1990–2023 time series to project absolute burden and age-standardized rates to 2050.

*Findings:* CKM-related DALYs increased from 3.82 million in 2000 to 8.71 million in 2023 (128%), and their share of all-cause DALYs rose from 20.2% to 25.0%. In 2023, premature mortality accounted for 76.8% of the burden; cardiovascular disease and type 2 diabetes together accounted for 88.4%, with diabetes predominating among adults aged 40–64 years. Mexico’s age-standardized DALY rate increased by 2.9% during 2000–2023, whereas rates declined by 16.1% in Latin America and the Caribbean and by 24.1% globally. Absolute DALYs are projected to reach 21.40 million by 2050 (95% prediction interval 19.27–23.77), while the age-standardized rate remains broadly unchanged.

*Conclusions:* Mexico has diverged from declining regional and global trends in age-standardized CKM-related burden. Persistent premature mortality, substantial diabetes burden during working age, and projected growth in absolute health loss support accelerated, coordinated public health and health-system action across the CKM continuum.

## Introduction

Metabolic risk factors, chronic kidney disease (CKD), and cardiovascular disease (CVD) are increasingly recognized as components of an interconnected pathophysiological and clinical continuum. The concept of cardiovascular–kidney–metabolic (CKM) health formalizes these relationships and emphasizes opportunities to prevent progression from excess or dysfunctional adiposity and metabolic risk to subclinical and clinical multiorgan disease [1]. Population-based evidence further indicates that the consequences of these conditions are cumulative: combinations of diabetes, CKD, and CVD are associated with progressively higher mortality and greater reductions in life expectancy than each condition considered separately [2, 3]. Metabolic dysfunction-associated steatotic liver disease (MASLD) is also closely connected to this continuum through shared metabolic determinants and is associated longitudinally with type 2 diabetes (T2D), cardiovascular events, CKD, and liver-related complications [4]. These interdependencies support assessing their population-level impact within a unified framework rather than exclusively as separate diseases.

CKM and related liver diseases represent a major and evolving source of health loss globally and across Latin America and the Caribbean [5]. However, their magnitude and trajectories vary substantially between countries. The burdens associated with obesity and diabetes have increased across much of the Americas, whereas the burden attributable to high systolic blood pressure has declined in several settings, illustrating that improvement in one CKM-related domain does not necessarily indicate progress across the broader CKM spectrum [6]. Differences in ethnic and social composition, population aging, metabolic risk exposure, epidemiological transition, and healthcare access can further shape country-specific patterns. Moreover, reductions in age-specific disability rates might not offset the increasing absolute burden generated by population growth and aging [7]. National analyses contextualized against regional and global patterns are therefore needed to identify divergences that might be obscured by broader geographical estimates.

Mexico provides a particularly relevant setting for such an assessment. Recent nationally representative surveys estimated that 37.1% of adults had obesity, 17.0% had T2D, and 29.1% had hypertension [8–10]. Complementary estimates from the Global Burden of Disease (GBD) Study indicate that metabolic risks accounted for 19.8% of total disability-adjusted life-years (DALYs) in 2021, with high fasting plasma glucose and high body-mass index among the leading contributors to health loss [11]. Disease-specific analyses have documented substantial and increasing burdens of CVD, T2D, and CKD [12–14]. The metabolic liver component has also increased: GBD-based estimates indicate that deaths due to MASLD rose by 229% between 1990 and 2023, although a nationally representative measured prevalence estimate remains unavailable [15]. Thus, the high prevalence of metabolic risk factors in Mexico is accompanied by substantial cardiovascular, kidney, metabolic, and related liver disease burden.

Despite this growing body of evidence, recent studies in Mexico have predominantly quantified individual risk factors or diseases separately. Their collective contribution to national health loss, the relative contributions of premature mortality and disability, and their evolution within a unified CKM-related framework remain insufficiently characterized. Furthermore, the future implications of the integrated burden have not been estimated, and Mexico’s trajectory has not been systematically contextualized against Latin American and Caribbean and global patterns. We therefore aimed to quantify the integrated and component-specific national burden of CKM-related diseases in Mexico from 2000 to 2023; examine their contributions to DALYs, years of life lost (YLLs), and years lived with disability (YLDs) and their temporal trends; compare Mexico’s trajectory with regional and global patterns; and project the future integrated CKM-related burden.

## Methods

### Study design and data sources

We conducted a population-level ecological time-series analysis using Global Burden of Diseases, Injuries, and Risk Factors Study 2023 (GBD 2023) estimates to characterize the magnitude, composition, age patterns, and temporal trajectory of CKM-related disease burden in Mexico. Descriptive, comparative, and temporal trend analyses covered 2000–2023. Forecasting analyses used annual estimates from 1990 to 2023 to generate projections for 2024–2050. Latin America and the Caribbean and global estimates were included as contextual comparators, and selected risk-attributable burdens were assessed separately.

GBD 2023 provides internally comparable annual estimates of mortality, morbidity, and health loss across 204 countries and territories [16]. Data were extracted from the GBD Results Tool on July 27, 2026. We retrieved DALYs, YLLs due to premature mortality, and YLDs, with corresponding 95% uncertainty intervals, for both sexes combined. Absolute and age-specific analyses were restricted to adults aged 20 years or older; age-standardized rates were obtained using the GBD “age-standardized” age category and were not restricted to this age range. Cause-specific estimates were used without alteration, whereas the integrated CKM-related construct and other derived measures were calculated as described below. The complete extraction parameters—including measure, metric, age, sex, cause, risk, location, and corresponding identifiers—are provided in Supplementary Table S1.

### Operational definition of the integrated CKM-related burden

Because GBD 2023 does not provide a dedicated cause category representing the CKM continuum, an operational definition was required. Previous population-level assessments have jointly analyzed selected CKM-related components, although their inclusion criteria and handling of disease and risk-attributable burden have varied [17–19]. We therefore prespecified a disease-based construct comprising mutually exclusive GBD cause categories grouped into four domains: CVD (ischemic heart disease, stroke, hypertensive heart disease, atrial fibrillation and flutter, and lower-extremity peripheral artery disease); kidney disease (CKD due to T2D or hypertension); metabolic disease (T2D); and metabolic liver disease. This aggregate quantifies population-level health loss and does not estimate CKM prevalence or individual-level multimorbidity.

For the hepatic domain, the GBD aggregate cause “Total burden related to Non-alcoholic fatty liver disease (NAFLD)” represented MASLD-related liver disease because GBD 2023 retains the historical nomenclature. The construct excluded type 1 diabetes; CKD not attributed to T2D or hypertension; cardiovascular causes not considered specific to the prespecified CKM pathways, including congenital and valvular disease; and alcohol-associated, viral, and other non-metabolic liver diseases.

For each year, cause-specific point estimates were summed within composite domains and then across the four domains, separately for DALYs, YLLs, and YLDs. Identical causes and aggregation rules were applied to Mexico, Latin America and the Caribbean, and globally. Exact GBD labels and identifiers, hierarchical levels, inclusion rationale, and handling are provided in Supplementary Table S2.

### Statistical analysis

Approximate 95% uncertainty intervals (UIs) for the composite cardiovascular and kidney domains were derived by inferring lower- and upper-side standard errors from the component GBD UIs and combining them separately through root-sum-of-squares propagation under an independence assumption. The equations and assumptions are provided in Supplementary Table S3. Original GBD UIs were retained unchanged for the single-cause T2D and NAFLD-related liver disease domains. No UIs were estimated for the integrated CKM-related aggregate or for proportions derived from it, including its share of all-cause DALYs and the relative contributions of individual domains, because component-level posterior draws and their covariance structure were unavailable.

The CKM-related share of total DALYs was calculated by dividing integrated CKM-related DALYs by all-cause DALYs within the same year and age range; domain contributions were calculated relative to the integrated aggregate. Relative changes between 2000 and 2023 were expressed as percentages, whereas changes in the CKM-related share were reported as both percentage-point differences and relative percentage changes. All aggregations and percentage changes were calculated from unrounded point estimates.

Temporal trends in annual age-standardized DALY rates during 2000–2023 were summarized using estimated annual percentage changes (EAPCs), derived from log-linear regression as 100×[exp(β)−1], where *β* represents the coefficient for calendar year. Trends were classified as increasing when the lower bound of the 95% CI exceeded zero and decreasing when the upper bound was below zero; otherwise, the EAPC was considered not statistically distinguishable from zero. These CIs quantify model-based uncertainty in the fitted trend and do not incorporate uncertainty in the underlying GBD estimates.

All-cause, all-age age-standardized DALY rates attributable to high body-mass index, high fasting plasma glucose, high systolic blood pressure, high low-density lipoprotein (LDL) cholesterol, smoking, kidney dysfunction, and low physical activity were examined separately as contextual indicators. Because risk-attributable estimates are not mutually exclusive, they were not summed or incorporated into the disease-based CKM aggregate.

Analyses were conducted in R version 4.4.2. Analytical code, derived datasets, extraction metadata, package versions, and the computational environment are available from the study’s GitHub repository (https://github.com/CKM-Mexico/ckm-burden-mexico) and permanently archived on Zenodo (version 1.0.0; https://doi.org/10.5281/zenodo.22694806). Reporting followed the Guidelines for Accurate and Transparent Health Estimates Reporting (GATHER; Supplementary material, pp. 6–9) [20].

### Forecasting analysis

Forecasting analyses used annual 1990–2023 estimates to project integrated CKM-related burden for 2024–2050.

Six series were modeled separately: absolute DALYs, YLLs, and YLDs among adults aged 20 years or older and the corresponding standard all-age GBD age-standardized rates.

Autoregressive integrated moving average (ARIMA) models were selected by exhaustive search using the corrected Akaike information criterion [21]. Absolute series were modeled on the log scale and back-transformed within the fable framework, using original-scale means as point forecasts; rates were modeled on their original scale.

Absolute series formed a hierarchy in which DALYs equaled YLLs plus YLDs; base forecasts were reconciled using MinT-shrink in fabletools [22]. Age-standardized rates were modeled independently.

Model adequacy was assessed with residual diagnostics, including the Ljung–Box test. Five-year holdout validation (training 1990–2018; evaluation 2019–2023) used RMSE, MAE, MAPE, and MASE on the original outcome scale. For absolute series, accuracy before and after reconciliation was compared. Model specifications, diagnostics, and validation results are reported in Supplementary Table S4.

Sensitivity analyses compared primary DALY forecasts with exponential smoothing state-space (ETS) models and, for the age-standardized rate, random walk with drift. For absolute DALYs, random walk with drift was mathematically equivalent to the selected unreconciled ARIMA(0,1,0) with drift and was not an independent alternative (Supplementary Table S5).

Final models were refitted to 1990–2023 and generated annual 2024–2050 forecasts. Point estimates and 80% and 95% prediction intervals were derived from 10,000 simulated trajectories; absolute trajectories were reconciled before interval extraction. These intervals represent conditional model uncertainty and exclude uncertainty in the underlying GBD estimates. Forecasts extrapolate historical patterns rather than demographic, risk-factor, treatment, or policy scenarios.

### Ethics approval

Ethics approval and informed consent were not required because the study used only aggregated, non-identifiable GBD 2023 estimates.

## Results

### Overall magnitude and contribution of CKM-related burden

Among adults aged 20 years or older, CKM-related DALYs increased from 3.82 million in 2000 to 8.71 million in 2023 (Figure 1), representing a 128% increase. Over the same period, all-cause DALYs increased from 18.90 million to 34.87 million (84.5%). The CKM-related share of total DALYs therefore rose from 20.2% to 25.0%, an increase of 4.8 percentage points or 23.5% in relative terms.

**Figure 1:**
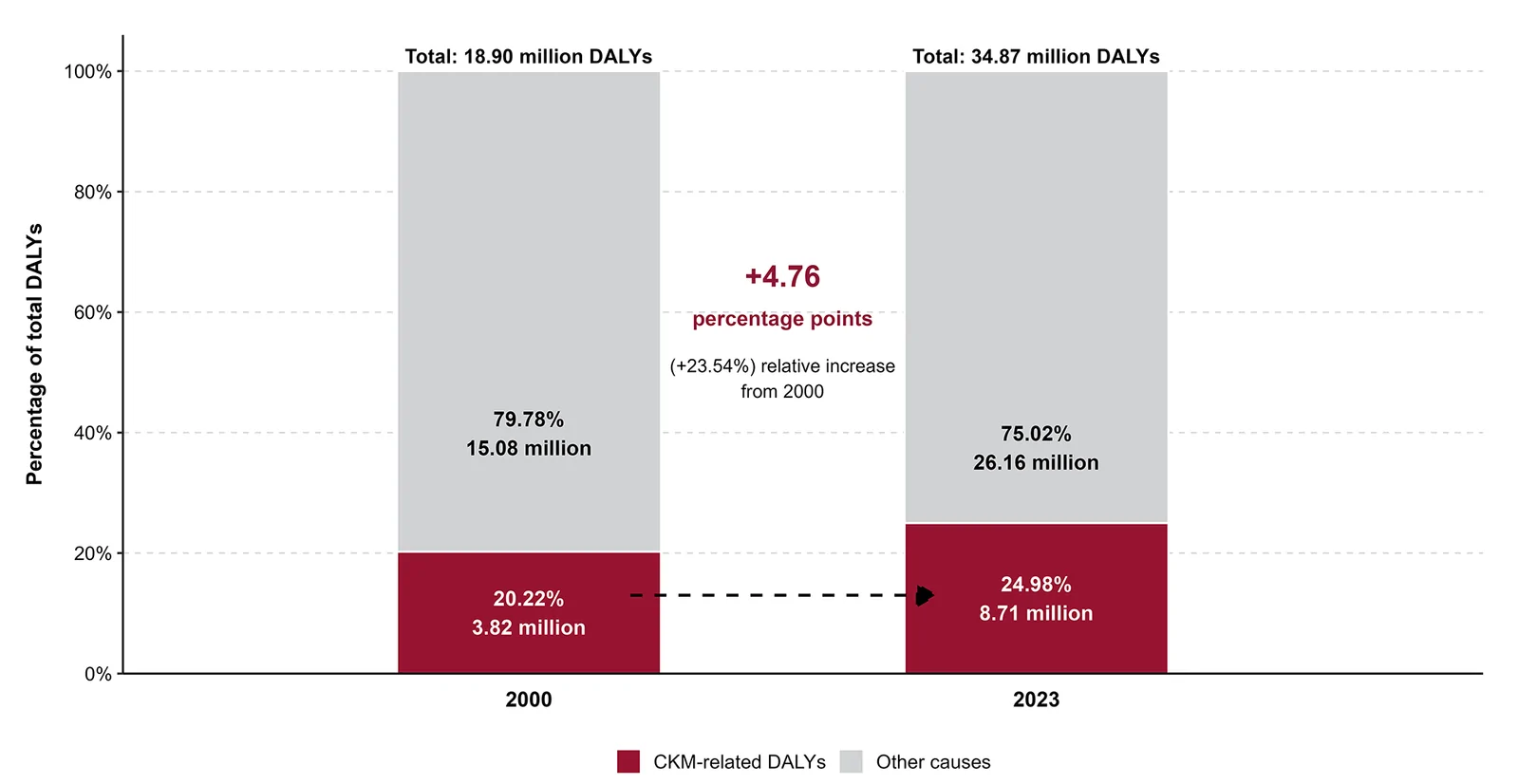
CKM-related share of total DALY burden among adults ≥ 20 years in Mexico, 2000 and 2023. Bars show absolute DALYs and their proportion of total all-cause DALYs among Mexican adults aged 20 years or older, both sexes combined. CKM-related burden includes the prespecified cardiovascular, kidney, type 2 diabetes, and MASLD-related liver disease domains; other causes were calculated as total DALYs minus CKM-related DALYs. Absolute change is expressed in percentage points, and relative change was calculated from unrounded proportions. CKM = cardiovascular–kidney–metabolic. DALY = disability-adjusted life-year. MASLD = metabolic dysfunction-associated steatotic liver disease.

YLLs increased from 2.9 million in 2000 to 6.69 million in 2023 (130.9%), whereas YLDs increased from 0.92 million to 2.02 million (118.5%). In the all-age age-standardized analyses, the YLL rate increased from 4,795.9 to 4,900.5 per 100,000 population (2.2%), and the YLD rate from 1,366.7 to 1,440.4 per 100,000 population (5.4%). In 2023, YLLs accounted for 76.8% of integrated CKM-related DALYs and YLDs for 23.2%.

### Composition and temporal trends of CKM-related burden

In 2023, the cardiovascular domain constituted the largest component of CKM-related burden, accounting for 3.94 million DALYs (45.2%), followed by T2D (3.76 million; 43.2%), kidney disease (0.73 million; 8.4%), and MASLD-related liver disease (0.27 million; 3.2%). Together, the cardiovascular and T2D domains accounted for 88.4% of CKM-related DALYs.

Crude CKM-related DALY rates increased steeply with age, from 1,518 per 100,000 population among adults aged 20–39 years to 10,674 among those aged 40–64 years and 35,390 among those aged 65 years or older (Figure 2A). CVD and diabetes jointly accounted for more than 90% of the burden in each age group, but their relative contributions varied with age (Figure 2B). The cardiovascular domain predominated among adults aged 20–39 years and those aged 65 years or older, whereas diabetes was the leading domain among adults aged 40–64 years, accounting for 51.9% of their CKM-related DALYs. The kidney contribution was greatest among older adults, while MASLD-related liver disease remained the smallest domain across age groups.

**Figure 2:**
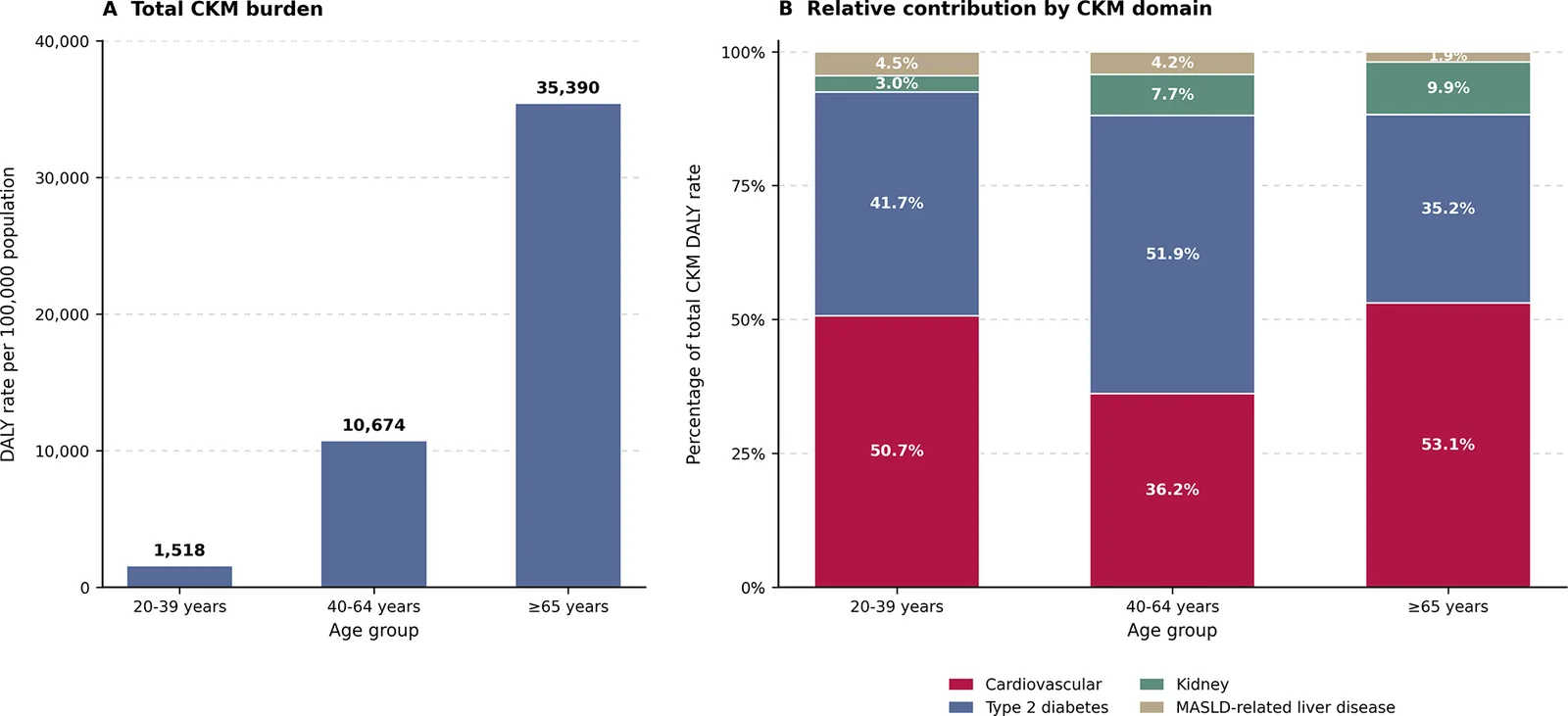
CKM-related disease burden by age group in Mexico, 2023. Panel **A** shows crude CKM-related DALY rates per 100,000 population by age group among Mexican adults, both sexes combined. Panel **B** shows the proportional contribution of each CKM domain to the total CKM-related DALY rate within each age group. The cardiovascular domain includes IHD, stroke, HHD, AF/AFL, and PAD; the kidney domain includes CKD due to T2D and hypertension. Percentages might not total 100% because of rounding. AF/AFL = atrial fibrillation and flutter. CKD = chronic kidney disease. CKM = cardiovascular–kidney–metabolic. DALY = disability-adjusted life-year. HHD = hypertensive heart disease. IHD = ischemic heart disease. MASLD = metabolic dysfunction-associated steatotic liver disease. PAD = peripheral artery disease. T2D = type 2 diabetes.

The four domains showed heterogeneous changes in age-standardized DALY rates between 2000 and 2023 (Table 1, Figure 3). The cardiovascular domain decreased by 5.7% overall, although its estimated annual trend was not statistically significant (EAPC 0.11, 95% CI −0.08 to 0.30). By contrast, the diabetes domain increased by 18.8% (EAPC 0.56%, 95% CI 0.32 to 0.81), whereas the kidney domain decreased by 12.4%, although its annual trend was not statistically significant (EAPC −0.54, 95% CI −1.10 to 0.02). The MASLD-related liver disease domain showed comparatively little overall change (5.3%) and no statistically significant annual trend (EAPC 0.10, 95% CI −0.11 to 0.31). Detailed cause-specific estimates and temporal changes are provided in Supplementary Table S6.

**Figure 3:**
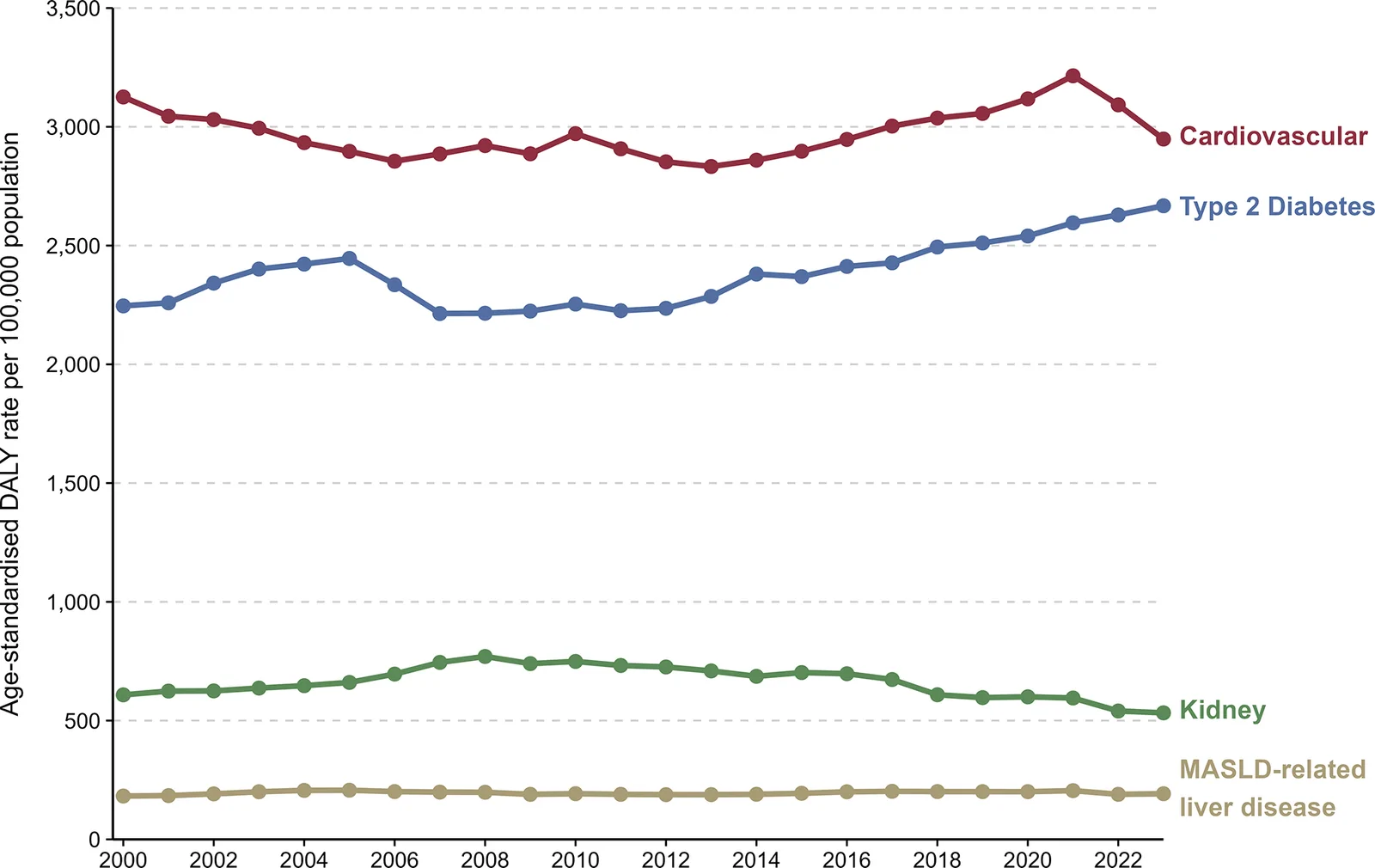
Temporal trends in CKM-related disease domains in Mexico, 2000–2023. The cardiovascular domain includes ischemic heart disease, stroke, hypertensive heart disease, peripheral artery disease, and atrial fibrillation and flutter; the kidney domain includes chronic kidney disease due to type 2 diabetes and hypertension. DALY = disability-adjusted life-year. CKM = cardiovascular–kidney–metabolic. MASLD = metabolic dysfunction-associated steatotic liver disease.

**Table 1:** CKM-related disease burden and temporal changes in Mexico, 2000–2023.

DISEASE	ABSOLUTE NUMBER, 2000 (95% UI)*	AGE-STANDARDIZED RATE PER 100,000, 2000 (95% UI)	ABSOLUTE NUMBER, 2023 (95% UI)*	AGE-STANDARDIZED RATE PER 100,000, 2023 (95% UI)	EAPC, 2000–2023 (95% CI)	CHANGE IN AGE-STANDARDIZED RATE, 2000–2023 (%)
**Cardiovascular**	1,868,708.53 (1,800,469.48 to 1,934,958.98)*	3,125.7 (3,013.0 to 3,233.1)	3,940,779.21 (3,786,813.07 to 4,066,072.92)*	2,948.6 (2,830.1 to 3,045.0)	0.11 (−0.08 to 0.30)	−5.7
**T2D**	1,465,723.4 (1,255,684.7 to 1,731,734.8)	2,246.1 (1,940.4 to 2,622.7)	3,762,848 (3,314,330.8 to 4,311,608.7)	2,667.6 (2,354.1 to 3,055.6)	0.56 (0.32 to 0.81)	18.8
**Kidney**	362,207.76 (313,676.32 to 420,517.09)*	608.3 (527.8 to 704.4)	731,439.89 (636,259.18 to 842,747.44)*	532.6 (463.7 to 610.5)	−0.54 (−1.10 to 0.02)	−12.4
**MASLD-related liver disease**	125,427.6 (88,927.4 to 168,589.8)	182.4 (129.0 to 244.7)	274,971.2 (202,934.1 to 357,599.5)	192.1 (143.4 to 249.1)	0.10 (−0.11 to 0.31)	5.3
**Integrated CKM-related burden**	3,822,067.42^†^	6,162.5^†^	8,710,038.56^†^	6,340.9^†^	0.22 (0.10 to 0.34)	2.9

*Single-cause domains retain the original GBD 95% uncertainty intervals; composite-domain intervals are approximate analytic 95% intervals derived by propagating cause-specific uncertainty (Supplementary file 1, Table S3). EAPC 95% confidence intervals reflect uncertainty in the fitted trends only. ^†^No uncertainty interval was calculated for the integrated CKM-related aggregate because posterior draws and cross-cause covariance were unavailable; point estimates sum the unrounded estimates of nine mutually exclusive causes. CI = confidence interval. CKM = cardiovascular–kidney–metabolic. EAPC = estimated annual percentage change. GBD = Global Burden of Disease. MASLD = metabolic dysfunction-associated steatotic liver disease.

### Temporal trends in all-cause disease burden attributable to selected CKM-relevant risk factors, 2000–2023

Age-standardized all-cause DALY rates attributable to selected risk factors relevant to CKM health showed heterogeneous changes between 2000 and 2023 (Figure 4). Rates attributable to high BMI, high fasting plasma glucose, and low physical activity increased by 20.1%, 9.8%, and 20.9%, respectively. By contrast, rates attributable to smoking, high systolic blood pressure, and kidney dysfunction decreased by 44.9%, 8.6%, and 7.4%, respectively, whereas the burden attributable to high LDL cholesterol showed comparatively little overall change (+6.0%). These estimates contextualize changes in the broader CKM risk environment and should not be interpreted as the direct or additive contribution of these risk factors to the integrated CKM-related burden.

**Figure 4:**
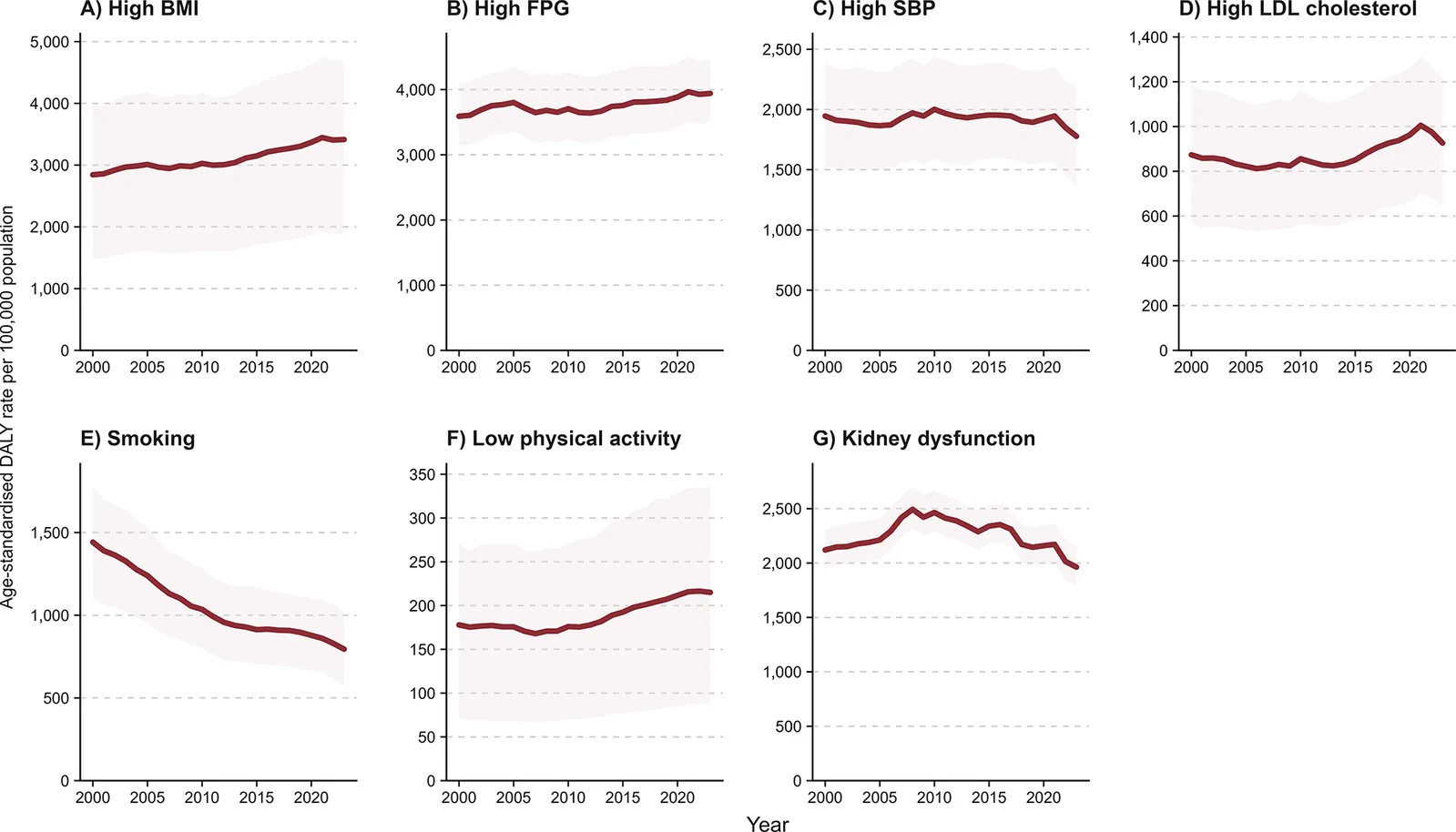
Temporal trends in all-cause disease burden attributable to selected CKM-relevant risk factors in Mexico, 2000–2023. Age-standardized DALY rates per 100,000 population, both sexes combined. Lines indicate point estimates and shaded areas 95% uncertainty intervals. Risk-attributable estimates are not mutually exclusive and should not be summed. BMI = body-mass index. CKM = cardiovascular–kidney–metabolic. DALY = disability-adjusted life-year. FPG = fasting plasma glucose. LDL = low-density lipoprotein. SBP = systolic blood pressure.

### Comparison with Latin America and the Caribbean and the global burden

Between 2000 and 2023, age-standardized integrated CKM DALY rates declined by 24.14% globally and by 16.13% in Latin America and the Caribbean, whereas the rate in Mexico increased by 2.89% (Figure 5). In 2000, Mexico had a substantially lower rate than the global estimate and a rate similar to that of Latin America and the Caribbean. By 2023, however, Mexico had the highest age-standardized CKM DALY rate (6,340.9 per 100,000 population), exceeding the global (5,511.6) and regional (5,065.2) estimates by 15.0% and 25.2%, respectively. These contrasting trends resulted in a widening disparity between Mexico and both comparator populations over the study period.

**Figure 5:**
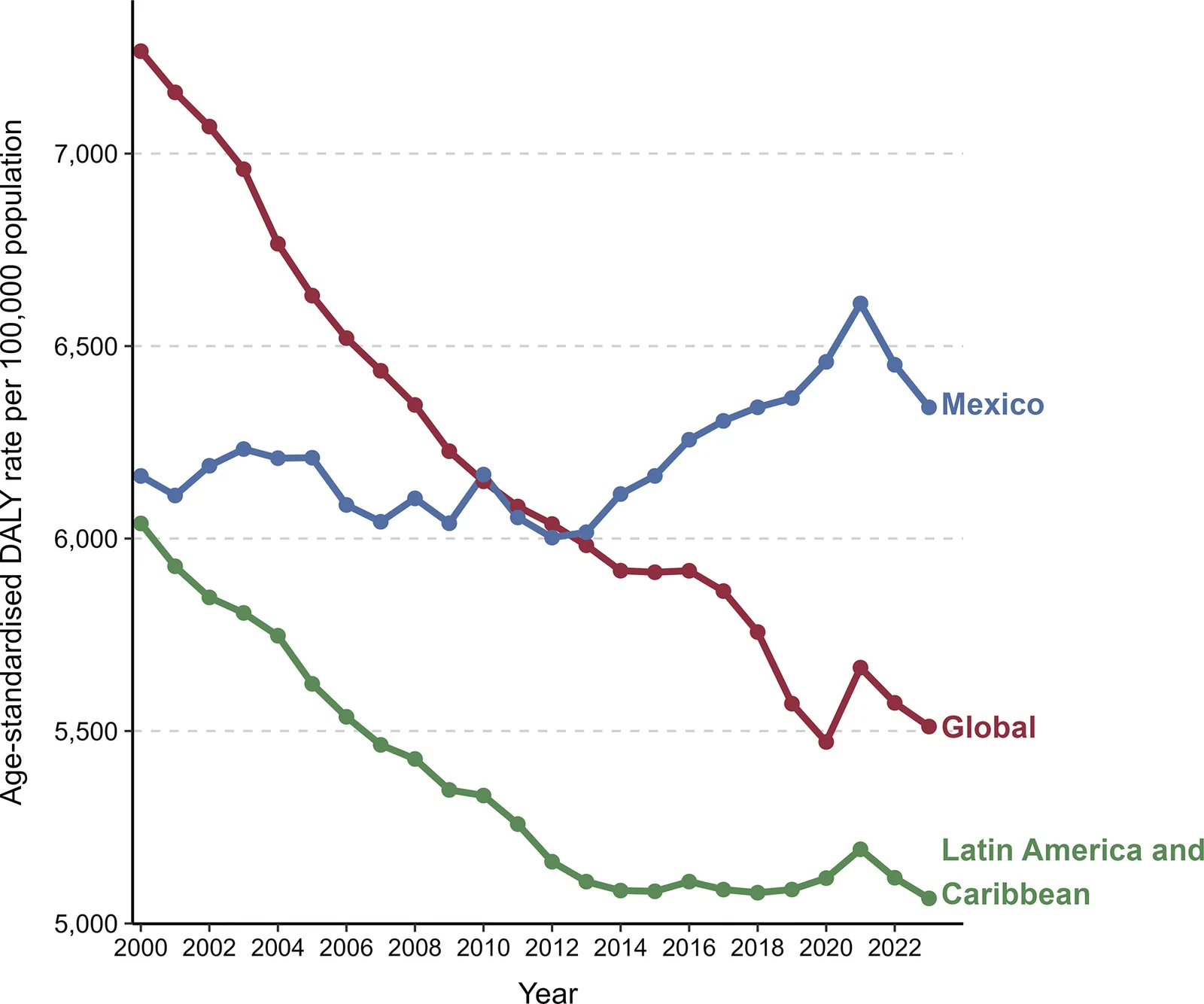
Temporal trends in the age-standardized integrated CKM-related DALY rate in Mexico, Latin America and the Caribbean, and globally, 2000–2023. Lines show annual point estimates per 100,000 population. Age-standardized rates were obtained directly from GBD 2023 using its standard all-age metric. The integrated CKM-related burden was operationally defined as the sum of mutually exclusive GBD cause categories allocated to cardiovascular, kidney, type 2 diabetes, and metabolic liver disease domains (Methods; Supplementary Table S2). DALY = disability-adjusted life-year. CKM = cardiovascular–kidney–metabolic. GBD = Global Burden of Diseases, Injuries, and Risk Factors Study.

### Changes in disease-specific CKM burden in Mexico, Latin America and the Caribbean, and globally

Changes in disease-specific age-standardized DALY rates between 2000 and 2023 differed across Mexico, Latin America and the Caribbean, and globally (Figure 6). T2D burden increased across all three geographies, although the increase was greater globally (23.1%) and in Mexico (18.8%) than in Latin America and the Caribbean (9.8%). Mexico showed a contrasting pattern for ischemic heart disease: its age-standardized DALY rate increased by 12.4%, whereas it decreased by 18.6% regionally and 27.1% globally. Stroke showed the largest decline across all three geographies, with reductions of similar magnitude in Mexico (38.0%), Latin America and the Caribbean (38.1%), and globally (39.4%). Hypertensive heart disease and peripheral artery disease also declined consistently, although the reductions were generally smaller in Mexico than globally. By contrast, the age-standardized burden of CKD due to T2D and hypertension decreased in Mexico by 13.5% and 11.3%, respectively, whereas both increased globally and changed comparatively little at the regional level. MASLD-related liver disease increased in Mexico (5.3%) and Latin America and the Caribbean (12.6%), but remained comparatively stable globally (–1.2%).

**Figure 6:**
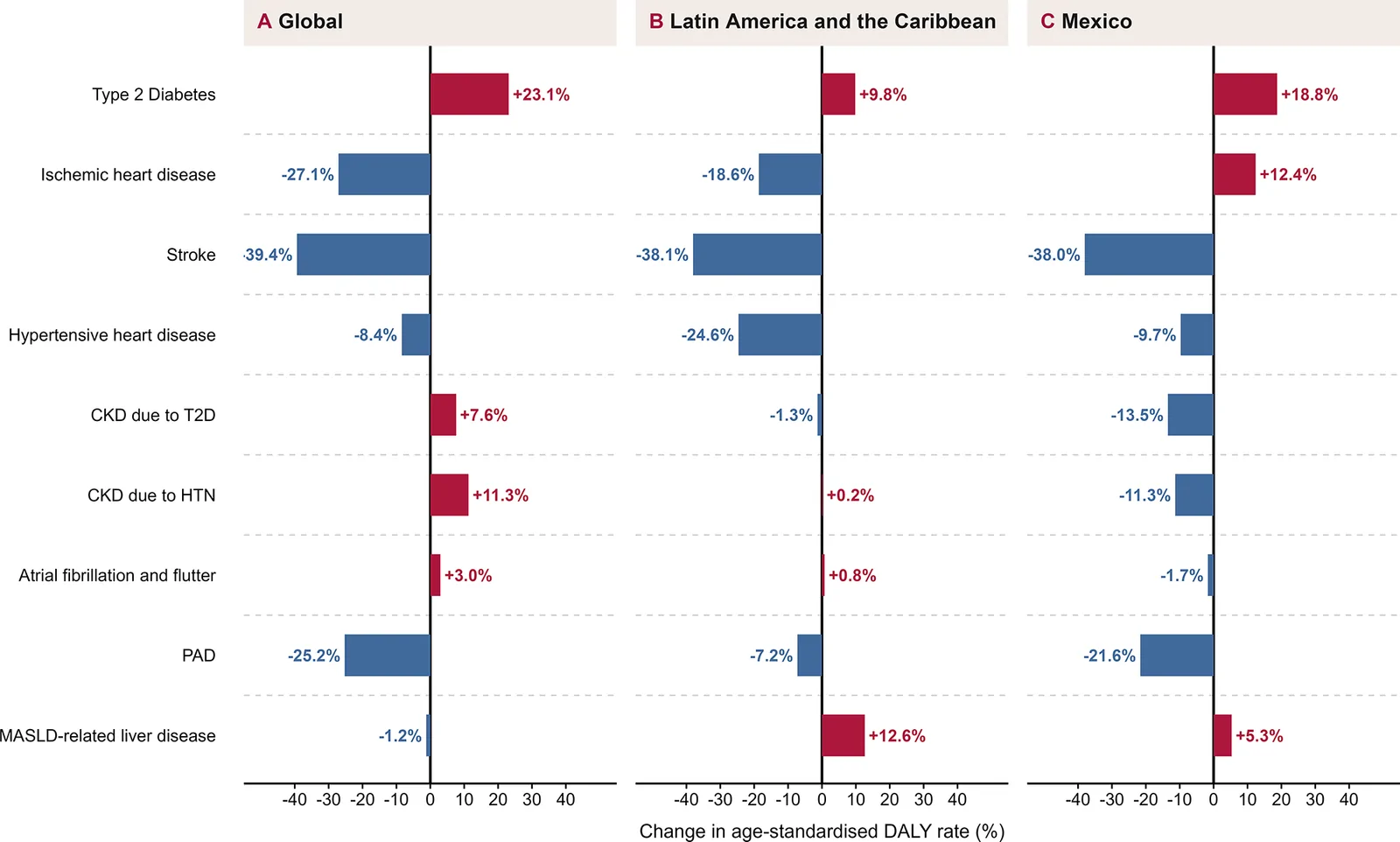
Percentage change in disease-specific age-standardized DALY rates for selected CKM-related causes in Mexico, Latin America and the Caribbean, and globally, 2000–2023. Percentage change in age-standardized DALY rates between 2000 and 2023, for both sexes combined, globally **(A)**, in Latin America and the Caribbean **(B)**, and in Mexico **(C)**. Red indicates an increase and blue indicates a decrease. CKD = chronic kidney disease. CKM = cardiovascular–kidney–metabolic. DALY = disability-adjusted life-year. GBD = Global Burden of Diseases, Injuries, and Risk Factors Study. HTN = hypertension. MASLD = metabolic dysfunction-associated steatotic liver disease. PAD = peripheral artery disease. T2D = type 2 diabetes.

### Projected CKM-related burden in Mexico

Five-year holdout MAPE ranged from 0.32% to 1.95% across the six principal series, and reconciliation preserved predictive accuracy (Supplementary Table S4). ETS and random-walk-with-drift sensitivity analyses yielded similar age-standardized DALY-rate projections: coefficients of variation were 0.38% in 2030 and 1.73% in 2050, with a maximum relative range of 3.03% (Supplementary Table S5).

Absolute CKM-related burden among adults aged ≥20 years was projected to increase substantially between 2024 and 2050 (Figure 7). Reconciled DALYs increased from 9.00 million in 2024 to 21.40 million in 2050, corresponding to a 137.9% increase. Absolute YLLs increased from 6.92 million to 16.37 million (136.4%), while absolute YLDs increased from 2.08 million to 5.03 million (142.6%) over the same period. YLLs remained the dominant component of projected health loss throughout the forecast period, accounting for approximately 76.5% of reconciled DALYs in 2050, whereas YLDs accounted for 23.5%.

**Figure 7:**
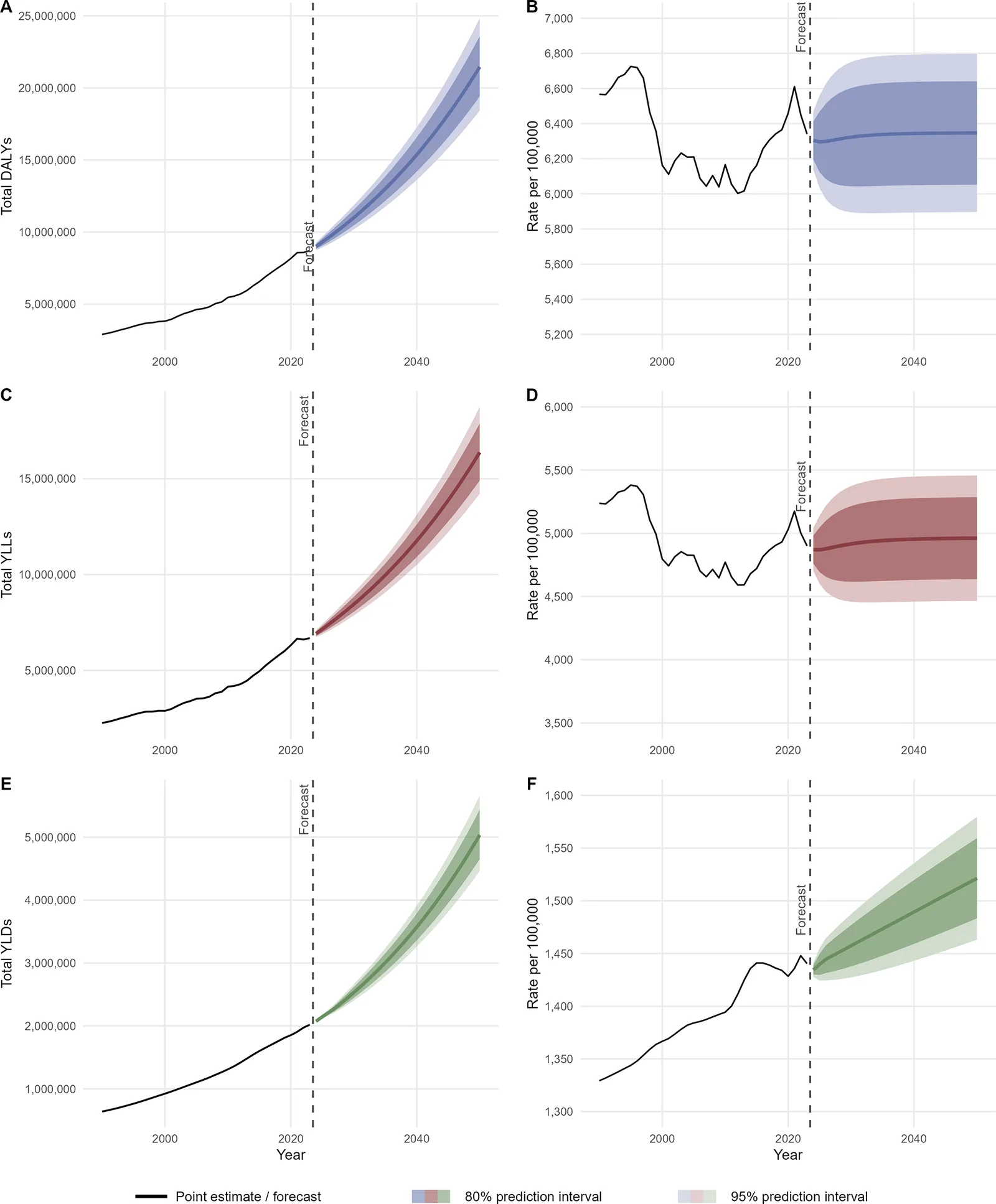
Observed and projected integrated CKM-related burden in Mexico, 1990–2050. **(A)** Total DALYs; **(B)** age-standardized DALY rate; **(C)** total YLLs; **(D)** age-standardized YLL rate; **(E)** total YLDs; and **(F)** age-standardized YLD rate. Black lines denote observed estimates (1990–2023), colored lines denote forecasts (2024–2050), darker shaded areas represent the 80% prediction interval, lighter shaded areas represent the 95% prediction interval, and the vertical dashed line marks the start of the forecast period. CKM = cardiovascular–kidney–metabolic. DALY = disability-adjusted life-year. YLLs = years of life lost. YLDs = years lived with disability.

Age-standardized DALY rates changed little, from 6,304 in 2024 to 6,347 per 100,000 in 2050 (0.7%); corresponding YLL and YLD rates increased by 1.8% and 6.1%, respectively. In 2050, the 95% prediction intervals were 19.27–23.77 million for reconciled DALYs and 5,902–6,800 per 100,000 for the age-standardized DALY rate (Supplementary Table S7).

## Discussion

In 2023, CKM-related diseases accounted for one quarter of DALYs among Mexican adults, predominantly through premature mortality. CVD and T2D contributed almost 90%, with T2D leading among adults aged 40–64 years. Although some components declined, Mexico’s age-standardized CKM-related DALY rate increased slightly, unlike regional and global rates, and absolute burden was projected to more than double by 2050. These findings indicate uneven progress, driven particularly by diabetes and ischemic heart disease.

Beyond documenting its magnitude, this study extends the application of the CKM framework from a predominantly clinical concept to the population-level assessment of health loss. The CKM framework was developed to emphasize the biological interdependence of metabolic risk, kidney disease, and CVD and the opportunities for prevention across their continuum [1]. Recent GBD-based assessments have quantified selected CKM components globally and regionally, but their operational definitions have varied—from constructs combining diseases and metabolic risk factors to disease-based constructs incorporating MASLD [17, 18]. Applying a prespecified set of CKM-related causes consistently across years and geographical settings provides an explicit, conceptually coherent, and reproducible approach to quantifying population health impact and permits consistent temporal and geographical comparisons. We deliberately restricted the definition to cardiovascular, kidney, diabetes, and liver disease categories aligned with the CKM framework, excluding congenital and valvular heart disease, glomerulonephritis and other or unspecified CKD, and alcohol-associated liver disease. The inclusion of MASLD-related liver disease extends this population-level construct to a closely related hepatic manifestation of metabolic dysfunction, supported by its shared metabolic determinants and associations with diabetes, CVD, and CKD [4]. That this focused construct still accounted for one quarter of total DALYs highlights the magnitude of health loss associated with interconnected CKM-related diseases. This aggregate quantifies population-level health loss from prespecified CKM-related causes; it does not estimate the prevalence of CKM syndrome or individual-level multimorbidity.

The predominance of YLLs indicates that Mexico’s CKM-related burden cannot be understood solely as longer survival with chronic disability in an aging population; it also entails substantial loss of life before the reference life expectancy. Although age-specific CKM-related DALY rates rose steeply with age, considerable health loss was already present during working age, and T2D was the leading domain among adults aged 40–64 years. This pattern could partly reflect early-onset diabetes and prolonged metabolic risk exposure. A previous GBD-based analysis showed that DALY rates from T2D among Mexican adolescents and young adults increased significantly between 1990 and 2019 [23]. Complementary evidence from national health survey 2018 showed that adults with early-onset diabetes had a longer duration since diagnosis and a higher proportion of macrovascular complications than those with later-onset disease [24]. Together, these observations are consistent with prolonged exposure to hyperglycemia and related metabolic abnormalities as a plausible contributor to the prominent diabetes burden during middle age. However, because the aggregated estimates used in our study do not capture age at diagnosis, disease duration, or individual disease trajectories, this interpretation remains hypothesis-generating.

The aggregate CKM trajectory concealed marked heterogeneity across its component diseases. Between 2000 and 2023, age-standardized DALY rates declined for stroke, peripheral arterial disease, and CKD due to T2D or hypertension, whereas rates for T2D and ischemic heart disease increased significantly. Mexico’s overall trajectory therefore does not indicate uniform deterioration, but rather that improvements in selected components were insufficient to offset the growing burden of diabetes and ischemic heart disease. The scale of diabetes was particularly striking: in 2023, its contribution approached that of the entire cardiovascular domain, whereas ischemic heart disease and stroke were the leading contributors in a recent global CKM assessment, albeit one using a different operational definition [17]. This pattern extends previous national GBD analyses documenting the prominent burden of T2D and CVD in Mexico [12, 13]. Conversely, the decline in DALY rates assigned to the two included CKD categories should not be interpreted as evidence of a low kidney disease burden or of broad improvement in CKD. Our operational definition excluded glomerulonephritis and other or unspecified CKD, which contribute substantially to Mexico’s total CKD burden [14].

Mexico’s divergence from regional and global trajectories was concentrated in specific components rather than observed across the entire CKM spectrum. Stroke declined by almost identical proportions in Mexico, Latin America and the Caribbean, and globally, indicating that the divergence was not universal. By contrast, the age-standardized burden of ischemic heart disease increased in Mexico while declining regionally and globally, and T2D increased across all three geographies. The accompanying risk-factor patterns provide a plausible, although non-causal, context: age-standardized all-cause DALY rates attributable to high body-mass index, high fasting plasma glucose, and low physical activity increased in Mexico; high LDL cholesterol increased modestly, whereas those attributable to smoking and high systolic blood pressure declined. This coexistence of favorable and adverse trends is consistent with an uneven risk-factor transition, in which progress in tobacco-related and blood-pressure-related burden has been insufficient to counter persistent metabolic risk. National surveys and GBD evidence similarly document a high prevalence of obesity and diabetes and a prominent contribution of metabolic risks to health loss in Mexico [8, 9, 11]. Because the risk-attributable estimates were all-cause, non-mutually exclusive, and analyzed separately from the derived CKM aggregate, they cannot establish that changes in these exposures caused the observed disease trajectories. Nevertheless, their convergence reinforces the prevention rationale for addressing excess adiposity, dysglycemia, and physical inactivity while sustaining gains in tobacco and blood-pressure control.

The forecasts extend the implications of the observed trends. Absolute CKM-related DALYs were projected to more than double between 2024 and 2050, while no meaningful decline in the age-standardized DALY rate was projected over the same period. These findings convey distinct information. The increase in absolute burden is compatible with population growth and aging, although their respective contributions cannot be quantified because our forecasting analysis did not include a demographic decomposition. Conversely, the absence of a decline in the age-standardized rate cannot be explained by population aging alone and indicates that, if recent trends persist, population-level health loss will not materially improve. Broader GBD forecasts anticipate declining all-cause age-standardized DALY rates in every super-region despite increasing DALY counts, largely because of population growth and aging [25]. Global CKM projections similarly show divergent trajectories across individual components, with declining age-standardized rates for ischemic heart disease and stroke but increasing rates for diabetes and high body-mass index. Mexico’s projected combination of rising absolute burden and no improvement in the age-standardized DALY rate therefore implies both increasing health-service needs and persistent epidemiological risk. However, ARIMA forecasts extrapolate historical patterns and do not incorporate demographic, risk-factor, treatment, or policy scenarios; projections are therefore conditional on the persistence of historical trends.

These findings point to complementary opportunities for action across the CKM continuum. At the population level, preventing the development and accumulation of metabolic risk should remain a priority, particularly through strategies addressing excess adiposity, dysglycemia, and physical inactivity, whose adverse temporal trends coincided with the increasing burden of T2D and ischemic heart disease in Mexico. Within health services, reducing established disease burden will require sustained achievement of evidence-based treatment goals for diabetes and hypertension, including comprehensive management of glycemia, blood pressure, lipids, and overall cardiovascular risk. Important gaps remain in the attainment of these goals and in key care processes, including continuity of follow-up and timely treatment intensification [26]. Earlier identification of kidney, cardiovascular, and metabolic liver involvement could create opportunities to prevent or delay progression, whereas timely evidence-based management of established and advanced complications remains essential to reduce premature mortality and disability. Routine monitoring of care processes, treatment-goal attainment, and clinical outcomes could help health systems translate the population-level burden documented here into targeted quality-improvement priorities [27].

This study has several strengths. It applied a prespecified set of mutually exclusive GBD causes consistently across 2000–2023 and across Mexico, Latin America and the Caribbean, and the global population, enabling temporal and geographical comparisons while minimizing double counting at the cause level. The combined assessment of DALYs, YLLs, YLDs, age-specific patterns, risk-attributable burden, and future trends provided complementary perspectives on the magnitude and composition of CKM-related health loss. The use of the complete 1990–2023 series for forecasting and reconciliation of the absolute DALY, YLL, and YLD forecasts further strengthened the internal coherence of the projected burden. Several limitations should nevertheless be considered. First, the findings inherit the limitations of GBD modeled estimates, including dependence on the availability and quality of source data, cause-of-death coding, redistribution procedures, and modeling assumptions; these limitations might be particularly relevant to the attribution of kidney and liver disease etiologies. Second, the integrated construct was investigator-defined and should not be regarded as a validated epidemiological definition of CKM syndrome. Alternative selections of component diseases would produce different estimates, and our focused definition does not represent the total burden of cardiovascular, kidney, or liver disease. Third, the aggregate quantifies health loss from diseases selected for their relevance to the CKM framework; it does not imply that all DALYs from these conditions are attributable to CKM syndrome. The included disease categories encompass heterogeneous etiologies, and the available estimates cannot distinguish the contributions of CKM-related and alternative etiological pathways. Furthermore, cause-specific DALYs cannot identify disease co-occurrence, individual trajectories, or interactions among CKM conditions; consequently, the aggregate does not quantify CKM syndrome prevalence or clinical multimorbidity. Fourth, because component-level posterior draws and covariance structures were unavailable, no uncertainty interval could be calculated for the integrated CKM-related aggregate or for proportions derived from it. Intervals for the composite cardiovascular and kidney domains relied on analytic approximations based on working assumptions of zero covariance and approximate normality and should not be interpreted as GBD posterior-draw-based uncertainty intervals. Confidence intervals for temporal trends and prediction intervals for forecasts likewise did not incorporate uncertainty in the underlying GBD estimates. Finally, the ecological design precludes causal inference regarding the correspondence between risk-attributable burden and disease-specific trajectories, and the forecasts extrapolate historical patterns without explicitly modeling future demographic, risk-factor, treatment, or policy scenarios.

## Conclusions

CKM-related diseases accounted for one quarter of health loss among Mexican adults in 2023, predominantly through premature mortality. Unlike regional and global patterns, Mexico showed no reduction in age-standardized burden, while absolute DALYs are projected to more than double by 2050 if historical trends persist. These findings support coordinated prevention of metabolic risk, improved treatment-goal attainment among people with diabetes and hypertension, earlier identification of organ involvement, and timely management of established complications, accompanied by continuous monitoring of care performance and population health outcomes.

## Data Availability

The original estimates analyzed in this study are available through the Institute for Health Metrics and Evaluation (IHME) Global Burden of Disease (GBD) Results Tool (https://vizhub.healthdata.org/gbd-results/), subject to the applicable IHME terms and Free-of-Charge Non-Commercial User Agreement. Data used for the original analyses were extracted on July 27, 2026. Equivalent queries were repeated on September 5–6, 2026, to assemble and validate the reproducibility repository. The original IHME exports are not redistributed. Detailed extraction parameters, including health outcomes, metrics, geographical locations, age groups, and study periods, are provided in Supplementary file 1 and the repository documentation. Analytical code, query metadata, derived analysis-ready datasets, canonical outputs, package versions, and validation records are publicly available from the GitHub repository (https://github.com/CKM-Mexico/ckm-burden-mexico). The definitive version 1.0.0 release is permanently archived on Zenodo (https://doi.org/10.5281/zenodo.22694806).

## Additional File

The additional file for this article can be found as follows:
