# Supplementary material for: Integrated Cardiovascular–Kidney–Metabolic Disease Burden in Mexico: Trends from 2000 to 2023 and Projections to 2050
Source: Ann Glob Health. 2026 Nov 10;92(1):111. doi: 10.5334/aogh.5558 (PMC13761797; doi:10.5334/aogh.5558)
Supplement: Supplementary file 1 [file agh-92-1-5558-s1.pdf]

SUPPLEMENTARY MATERIAL

1. **Table S1:** GBD 2023 extraction parameters used in the analysis.

2. **Table S2:** GBD 2023 causes included in the operational CKM-related construct and selected risks used for contextual analyses.
3. **Derivation of approximate analytic intervals for composite CKM domains.** **Table S3:** Computational procedure used to derive approximate analytic 95% intervals for composite CKM domains.
4. **GATHER checklist:** Completed Guidelines for Accurate and Transparent Health Estimates Reporting checklist.

5. **Table S4:** Specifications, diagnostic results, and validation performance of the forecasting models.
6. **Table S5:** Sensitivity analyses of projected integrated CKM-related DALY burden in Mexico.
7. **Table S6:** Cause-specific CKM-related disease burden and temporal changes in Mexico, 2000–23.
8. **Table S7.** Projected CKM-related burden in Mexico, selected years, 2024–50.

Table S1. GBD 2023 extraction parameters and analytical scope.

Analysis	Measure	Metric	Age	Sex	Geography	Cause/risk	Relevant IDs	Years
Overall CKM magnitude	DALYs, YLLs, YLDs	Number	Age-specific groups from 20–24 years onwards, aggregated to ≥20 years	Both sexes	Mexico	Nine CKM-related causes	Cause IDs: Table S2	2000, 2023
Share of total burden	DALYs	Number	Age-specific groups from 20–24 years onwards, aggregated to ≥20 years	Both sexes	Mexico	All causes and nine CKM-related causes	Cause IDs: Table S2	2000, 2023
Age-specific analysis	DALYs; population estimates where rates were derived	Number; rate where applicable	Age-specific groups aggregated to 20–39, 40–64, and ≥65 years	Both sexes	Mexico	Nine CKM-related causes	Cause IDs: Table S2	2023
Temporal trends	DALYs	Rate	Age-standardised (standard all-age GBD metric)	Both sexes	Mexico	CKM domains and component causes	Cause IDs: Table S2	2000–23
Geographical comparison	DALYs	Rate	Age-standardised (standard all-age GBD metric)	Both sexes	Mexico; Latin America and Caribbean; global	Nine CKM-related causes	Cause IDs: Table S2	2000–23
Risk-factor context	DALYs	Rate	Age-standardised (standard all-age GBD metric)	Both sexes	Mexico	Seven selected risks; all-cause attributable burden	Risk IDs: Table S2	2000–23
Forecasting: absolute burden	DALYs, YLLs, YLDs	Number	Age-specific groups from 20–24 years onwards, aggregated to ≥20 years	Both sexes	Mexico	Nine CKM-related causes; integrated aggregate derived analytically	Cause IDs: Table S2	1990–2023
Forecasting: age-standardised rates	DALYs, YLLs, YLDs	Rate	Age-standardised (standard all-age GBD metric)	Both sexes	Mexico	Nine CKM-related causes; integrated aggregate derived analytically	Cause IDs: Table S2	1990–2023

CKM=cardiovascular–kidney–metabolic. DALY=disability-adjusted life-year. GBD=Global Burden of Disease. YLD=year lived with disability. YLL=year of life lost.

Table S2. GBD 2023 causes included in the operational CKM-related construct and selected risks used for contextual analyses.

Panel A. Causes included in the integrated CKM-related aggregate

CKM domain	Exact GBD cause label	GBD cause ID	GBD hierarchy level	Study rationale for inclusion	Analytical and hierarchical handling
Cardiovascular	Ischemic heart disease	493	3	Major atherosclerotic cardiovascular manifestation within the CKM framework	Included directly as a most-detailed GBD cause
Cardiovascular	Stroke	494	3	Major cerebrovascular manifestation within the CKM framework	Included at the parent-cause level; stroke subtypes were not added separately
Cardiovascular	Hypertensive heart disease	498	3	Cardiovascular manifestation associated with sustained exposure to high blood pressure	Included directly as a most-detailed GBD cause
Cardiovascular	Atrial fibrillation and flutter	500	3	Clinically relevant arrhythmic cardiovascular manifestation associated with metabolic and cardiovascular disease	Included directly as a most-detailed GBD cause
Cardiovascular	Lower extremity peripheral arterial disease	502	3	Major manifestation of systemic atherosclerotic cardiovascular disease	Included directly as a most-detailed GBD cause
Kidney	Chronic kidney disease due to hypertension	591	4	Kidney disease attributed by GBD to a hypertensive pathway relevant to the CKM continuum	Included directly; the parent chronic kidney disease category was not included
Kidney	Chronic kidney disease due to diabetes mellitus type 2	998	4	Kidney disease attributed by GBD to type 2 diabetes and directly relevant to the CKM continuum	Included directly; the parent chronic kidney disease category was not included
Metabolic	Diabetes mellitus type 2	976	4	Core metabolic disease within the CKM framework	Included directly; the parent diabetes mellitus category was not included
Hepatic	Total burden related to Non-alcoholic fatty liver disease (NAFLD)	1028	1*	Operational GBD representation of metabolically related liver disease and the closest available proxy for MASLD	Included as a single special GBD cause category; alcohol-associated and other non-metabolic liver diseases were not included

Panel B. Selected GBD risks used only for contextual analyses

Exact GBD risk label	GBD risk ID	GBD hierarchy level	Parent risk category	Study rationale	Analytical handling
High body-mass index	108	2	Metabolic risks	Represents excess adiposity, an upstream component of CKM risk	Analysed separately using all-cause age-standardised DALY rates
High fasting plasma glucose	105	2	Metabolic risks	Represents dysglycaemia and metabolic dysfunction relevant to CKM progression	Analysed separately using all-cause age-standardised DALY rates
High systolic blood pressure	107	2	Metabolic risks	Major shared determinant of cardiovascular and kidney disease	Analysed separately using all-cause age-standardised DALY rates
High LDL cholesterol	367	2	Metabolic risks	Represents atherogenic lipid-related cardiovascular risk	Analysed separately using all-cause age-standardised DALY rates
Smoking	99	3	Tobacco; Behavioral risks	Major behavioural cardiovascular risk and relevant comparator for the evolving risk environment	Analysed separately using all-cause age-standardised DALY rates
Kidney dysfunction	341	2	Metabolic risks	Captures health loss attributable to impaired kidney function within the GBD risk framework	Analysed only as a risk-attributable estimate and not as a CKM disease component
Low physical activity	125	2	Behavioral risks	Behavioural risk associated with adverse metabolic and cardiovascular health	Analysed separately using all-cause age-standardised DALY rates

Note. GBD cause ID corresponds to cause_id, and GBD risk ID corresponds to rei_id in the exported GBD data. Exact GBD labels were retained in this table. Hierarchy levels were determined from the GBD 2023 cause and risk hierarchies.

*"Total burden related to Non-alcoholic fatty liver disease (NAFLD)" is classified as a level 1 category because it appears as a direct child of "All causes" in the GBD 2023 hierarchy. It represents a special reporting category and should not be interpreted as equivalent in scope to the three conventional broad level 1 cause groups.

Abbreviations: CKM=cardiovascular–kidney–metabolic. DALY=disability-adjusted life-year. GBD=Global Burden of Diseases, Injuries, and Risk Factors Study. LDL=low-density lipoprotein. MASLD=metabolic dysfunction-associated steatotic liver disease. NAFLD=non-alcoholic fatty liver disease.

Verification source: GBD 2023 Cause, REI, and Location Hierarchies and GBD Results Tool metadata, accessed Jul 27, 2026.

Derivation of approximate analytic intervals for composite CKM domains

For each component cause i, $\hat{\theta}_i$ denotes the GBD point estimate, and L_i and U_i denote the lower and upper bounds of its 95% uncertainty interval. Approximate intervals for each composite domain D were calculated as shown in Table S3.

The procedure was applied separately to absolute DALY numbers and age-standardised DALY rates for the cardiovascular and kidney domains. It assumed zero covariance among component estimation uncertainties and approximate normality on each side of the point estimate. Original GBD uncertainty intervals were retained for the single-cause type 2 diabetes and NAFLD-related liver disease domains. No interval was calculated for the integrated CKM-related aggregate or derived proportions because component-level posterior draws and covariance structures were unavailable. Composite-domain intervals should not be interpreted as GBD posterior-draw-based uncertainty intervals.

Table S3. Computational procedure used to derive approximate analytic 95% intervals for composite CKM domains.

Step	Calculation	Description
Component lower-side standard error	$SE_{i,L} = \frac{\hat{\theta}_i - L_i}{1.96}$	Approximates uncertainty below the point estimate for component cause (i).
Component upper-side standard error	$SE_{i,U} = \frac{U_i - \hat{\theta}_i}{1.96}$	Approximates uncertainty above the point estimate for component cause (i).
Composite point estimate	$\widehat{\theta_D} = \sum_{i=1}^{k} \hat{\theta}_i$	Sums the point estimates of the mutually exclusive causes included in domain (D).
Composite lower-side standard error	$SE_{D,L} = \sqrt{\sum_{i=1}^{k} {SE_{i,L}}^2}$	Propagates lower-side uncertainty using the root-sum-of-squares method.
Composite upper-side standard error	$SE_{D,U} = \sqrt{\sum_{i=1}^{k} {SE_{i,U}}^2}$	Propagates upper-side uncertainty using the root-sum-of-squares method.
Approximate lower bound	$L_D = \widehat{\theta_D} - 1.96 \times SE_{D,L}$	Defines the lower bound of the approximate analytic 95% interval.
Approximate upper bound	$U_D = \widehat{\theta_D} + 1.96 \times SE_{D,U}$	Defines the upper bound of the approximate analytic 95% interval.

GATHER checklist

Integrated cardiovascular–kidney–metabolic disease burden in Mexico: trends from 2000 to 2023 and projections to 2050

Prepared according to the Guidelines for Accurate and Transparent Health Estimates Reporting (GATHER).

#	GATHER checklist item	Description of compliance	Reference
**Objectives and funding**			
1	Define the indicator(s), populations (including age, sex, and geographic entities), and time period(s) for which estimates were made.	Reported. DALYs, YLLs, YLDs, absolute numbers, age-specific and age-standardised rates, populations, geography, and study periods are defined.	Introduction (final paragraph); Methods, sections 2.1–2.4.
2	List the funding sources for the work.	Reported. The manuscript states that the study received no funding and describes the authors' responsibility for the analysis and submission decision.	Methods, section 2.6 (Role of the funding source).
**Data inputs**			
For all data inputs from multiple sources that are synthesised as part of the study:			
3	Describe how the data were identified and how the data were accessed.	Reported. GBD 2023 is identified as the data source. The manuscript, appendix, and repository document the IHME GBD Results Tool access route, extraction period, query dimensions, selected causes and risks, and validation procedures.	Methods, sections 2.1–2.2; Data availability; appendix Table S1; study repository (doi:10.5281/zenodo.22694806).
4	Specify the inclusion and exclusion criteria. Identify all ad-hoc exclusions.	Reported. The prespecified disease-based CKM construct, inclusion logic, mutually exclusive cause hierarchy, exclusions, and rationale are described.	Methods, section 2.2; appendix Table S2.

#	GATHER checklist item	Description of compliance	Reference
5	Provide information about all included data sources and their main characteristics. For each data source used, report reference information or contact name/institution, population represented, data collection method, year(s) of data collection, sex and age range, diagnostic criteria or measurement method, and sample size, as relevant.	Reported. The immediate analytical source is GBD 2023. The manuscript and appendix document the principal characteristics of the exported estimates, including location, sex, age, cause, measure, metric, year, and uncertainty fields.	Methods, sections 2.1–2.2; appendix Tables S1–S2; cited GBD 2023 publications and GHDx documentation.
6	Identify and describe any categories of input data that have potentially important biases (eg, based on characteristics listed in item 5).	Reported. Potential biases arising from heterogeneous source-data availability and quality, cause-of-death attribution, non-fatal modelling, and cross-location comparability are discussed.	Discussion (strengths and limitations); cited GBD 2023 publications and appendices.
For data inputs that contribute to the analysis but were not synthesised as part of the study:			
7	Describe and give sources for any other data inputs.	Reported. No external covariates or non-GBD analytical datasets were used. Population denominators, geographical groupings, cause estimates, and risk-attributable estimates were obtained from GBD 2023.	Methods, sections 2.1 and 2.3; appendix Table S1.
For all data inputs:			
8	Provide all data inputs in a file format from which data can be efficiently extracted (eg, a spreadsheet rather than a PDF), including all relevant metadata listed in item 5. For any data inputs that cannot be shared because of ethical or legal reasons, such as third-party ownership, provide a contact name or the name of the institution that retains the right to the data.	Reported. Derived analytical datasets and the metadata required to reconstruct the study inputs are available in machine-readable formats. Original GBD estimates remain accessible through the IHME GBD Results Tool, subject to its applicable terms of use.	Data availability; appendix Tables S1–S2; study repository (doi:10.5281/zenodo.22694806).
Data analysis			
9	Provide a conceptual overview of the data analysis method.	Reported. The analysis is organised as extraction and validation, construction of mutually exclusive CKM domains, descriptive estimation, trend analysis, forecasting, reconciliation, and sensitivity analysis.	Methods, sections 2.1–2.4; appendix Tables S1–S5.

#	GATHER checklist item	Description of compliance	Reference
10	Provide a detailed description of all steps of the analysis, including mathematical formulae. This description should cover, as relevant, data cleaning, data pre-processing, data adjustments and weighting of data sources, and mathematical or statistical model(s).	Reported. Cause selection, aggregation, derived proportions and changes, EAPC regression, approximate interval calculations, ARIMA forecasting, bias-adjusted back-transformation, forecast reconciliation, hold-out validation, and sensitivity models are described. Executable scripts implement these steps.	Methods, sections 2.2–2.4; appendix Tables S2–S5; repository code and documentation.
11	Describe how candidate models were evaluated and how the final model(s) were selected.	Reported. Candidate ARIMA models were searched exhaustively and selected by AICc; adequacy was assessed using residual diagnostics and a five-year hold-out. ETS and random walk with drift were used as alternative models.	Methods, section 2.4; appendix Tables S4-S5.
12	Provide the results of an evaluation of model performance, if done, as well as the results of any relevant sensitivity analysis.	Reported. Forecast specifications, residual diagnostics, and five-year hold-out RMSE, MAE, MAPE, and MASE are reported. Sensitivity analyses compare the primary ARIMA forecasts with ETS and random walk with drift models.	Results, section 3.6; appendix Tables S4-S5.
13	Describe methods of calculating uncertainty of the estimates. State which sources of uncertainty were, and were not, accounted for in the uncertainty analysis.	Reported. The manuscript distinguishes GBD uncertainty intervals, EAPC confidence intervals, and forecast prediction intervals, and states which sources of uncertainty were not propagated. No uncertainty interval was calculated for the integrated disease aggregate because posterior draws and cross-cause covariance information were unavailable.	Methods, sections 2.3–2.4; appendix Tables S3 and S7; Discussion (limitations).
14	State how analytical or statistical source code used to generate estimates can be accessed.	Reported. Complete analytical code, the master execution script, package versions, renv.lock, data dictionaries, and execution and validation instructions are publicly accessible.	Methods, section 2.3; Data availability; study repository (doi:10.5281/zenodo.22694806).
Results and discussion			
15	Provide published estimates in a file format from which data can be efficiently extracted.	Reported. Machine-readable files provide the descriptive estimates, trend results, validation metrics, sensitivity analyses, forecasts, and data underlying the manuscript and appendix tables and figures.	Main tables and figures; appendix Tables S4–S7; study repository (doi:10.5281/zenodo.22694806).

#	GATHER checklist item	Description of compliance	Reference
16	Report a quantitative measure of the uncertainty of the estimates (eg, uncertainty intervals).	Reported. Cause- and domain-specific estimates include 95% uncertainty intervals where available; EAPCs include 95% confidence intervals; and forecasts include 80% and 95% prediction intervals. The integrated disease aggregate is reported without an uncertainty interval, with the reason stated explicitly.	Methods, sections 2.3–2.4; main Table 1 and Figure 4; appendix Tables S3, S6, and S7.
17	Interpret results in light of existing evidence. If updating a previous set of estimates, describe the reasons for changes in estimates.	Reported. Findings are interpreted against CKM, disease-specific, regional, and global evidence. This study is not an update of an estimate previously produced by the authors.	Introduction; Discussion; Research in Context.
18	Discuss limitations of the estimates. Include a discussion of any modelling assumptions or data limitations that affect interpretation of the estimates.	Reported. Limitations cover GBD input data and modelling, the investigator-defined CKM construct, lack of individual-level multimorbidity information, cause attribution, aggregation, uncertainty propagation, forecasting assumptions, and causal interpretation.	Discussion (strengths and limitations); Methods, sections 2.2–2.4.

Table S4. Forecast specifications, diagnostics, and five-year holdout performance.

Panel A. Final model specifications and residual diagnostics

Measure	Scale	Selected ARIMA model	AICc	Ljung–Box Q (df); p value
DALYs	Absolute	ARIMA(0,1,0) + drift	−182·88	7·81 (10); 0·647
DALYs	Age-standardised rate	ARIMA(2,0,0) + mean	406·84	5·23 (8); 0·733
YLLs	Absolute	ARIMA(0,1,0) + drift	−165·03	6·96 (10); 0·729
YLLs	Age-standardised rate	ARIMA(2,0,0) + mean	408·09	4·85 (8); 0·773
YLDs	Absolute	ARIMA(0,1,4) + drift	−280·67	1·50 (6); 0·960
YLDs	Age-standardised rate	ARIMA(0,1,3) + drift	184·97	2·46 (7); 0·930

Panel B. Five-year holdout validation, 2019–2023

Measure	Scale	Forecast	RMSE	MAE	MAPE	MASE
DALYs	Absolute	Base	152 303	128 065	1·50%	0·774
DALYs	Absolute	MinT-shrink reconciled	153 999	129 866	1·53%	0·785
YLLs	Absolute	Base	152 637	120 141	1·84%	0·955
YLLs	Absolute	MinT-shrink reconciled	149 385	117 584	1·80%	0·935
YLDs	Absolute	Base	45 938	38 238	1·95%	0·956
YLDs	Absolute	MinT-shrink reconciled	45 936	38 235	1·95%	0·956
DALYs	Age-standardised rate	Independent	114·62	85·53	1·31%	1·300
YLLs	Age-standardised rate	Independent	113·46	84·56	1·66%	1·282
YLDs	Age-standardised rate	Independent	5·56	4·53	0·32%	1·088

Panel A reports the final models selected after fitting the complete 1990–2023 series. Ljung–Box tests used lag 10; degrees of freedom account for the fitted autoregressive and moving-average terms. Panel B reports five-year hold-out validation using 1990–2018 as the training period and 2019–23 as the evaluation period. RMSE and MAE are expressed as numbers of DALYs, YLLs, or YLDs for the absolute series and as units per 100 000 population for the age-standardised rates. MAPE is expressed as a percentage and MASE is unitless. Absolute-series models were fitted on the logarithmic scale; their forecasts were back-transformed, and all hold-out accuracy metrics were calculated on the original outcome scale. Absolute forecasts are shown before and after MinT-shrink reconciliation. Age-standardised rate series were modelled independently and were not reconciled. AICc=corrected Akaike information criterion. ARIMA=autoregressive integrated moving average. DALY=disability-adjusted life-year. MAE=mean absolute error. MAPE=mean absolute percentage error. MASE=mean absolute scaled error. RMSE=root mean squared error. YLD=year lived with disability. YLL=year of life lost.

Table S5. Sensitivity analyses of projected integrated CKM-related DALY burden in Mexico.

Outcome	Model	2024	2030	2050	Change, 2024–50	Interpretive role
DALYs, absolute number (age ≥20 years)	Primary ARIMA (final manuscript forecast)	8,997,435	10,982,236	21,401,415	137.9%	Primary comparison
DALYs, absolute number (age ≥20 years)	ETS(M,A,N)	9,002,923	10,979,025	21,275,924	136·3%	Independent alternative
DALYs, absolute number (age ≥20 years)	Random walk with drift	9,005,766	11,004,775	21,502,383	138·8%	Equivalent to primary ARIMA(0,1,0) with drift before reconciliation
DALYs, age-standardised rate per 100,000	Primary ARIMA (final manuscript forecast)	6,304·0	6,322·9	6,346·6	0·7%	Primary comparison
DALYs, age-standardised rate per 100,000	ETS(M,N,N)	6,340·9	6,340·9	6,340·9	0·0%	Independent alternative
DALYs, age-standardised rate per 100,000	Random walk with drift	6,334·0	6,292·9	6,156·0	-2·8%	Independent alternative

Absolute numbers refer to adults aged 20 years or older; age-standardised rates are expressed per 100,000 population. Primary ARIMA values are the final manuscript forecasts. ETS and random-walk estimates are sensitivity estimates. For absolute DALYs, random walk with drift is mathematically equivalent to the selected ARIMA(0,1,0) with drift before reconciliation and is not an independent alternative.

Table S6. Cause-specific CKM-related disease burden and temporal changes in Mexico, 2000–2023.

Disease	Absolute number, 2000 (95% UI)	Age-standardised rates per 100,000; 2000 (95% UI)	Absolute number, 2023 (95% UI)	Age-standardised rates per 100,000; 2023 (95% UI)	Estimated Annual Percent Change 2000-2023	Overall Change 2000-2023 (%)
T2D	1,465,723.4 (1,255,684.7 to 1,731,734.8)	2246.1 (1940.4 to 2622.7)	3,762,848 (3,314,330.8 to 4,311,608.7)	2667.6 (2354.1 to 3055.6)	0.56 (0.32 to 0.81)	18.8
IHD	1,108,256.3 (1,053,141 to 1,154,615.4)	1828.1 (1738.6 to 1902.5)	2,772,213 (2,632,530.9 to 2,875,176.8)	2055.2 (1947.8 to 2134.8)	0.90 (0.66 to 1.15)	12.4
Stroke	600,490 (563,161 to 643,487)	1008.4 (945.5 to 1078.0)	815,897 (759,589.1 to 870,786.9)	625.2 (581.4 to 667.6)	-1.73 (-1.85 to -1.61)	-38.0
HHD	84,911.7 (77,136.9 to 91,898.8)	151.8 (137.9 to 164.2)	180,672.1 (166,668.8 to 195,926)	137.1 (126.4 to 148.6)	0.13 (-0.16 to 0.42)	-9.7
AF/AFL	64,232.4 (51,869.2 to 81,960.8)	117.3 (95.1 to 147.8)	151,592.6 (123,733.8 to 192,984)	115.4 (94.4 to 146.3)	0.02 (-0.04 to 0.09)	-1.7
MASLD-related liver disease	125,427.6 (88,927.4 to 168,589.8)	182.4 (129.0 to 244.7)	274,971.2 (202,934.1 to 357,599.5)	192.1 (143.4 to 249.1)	0.10 (-0.11 to 0.31)	5.3
PAD	10,817 (7,333.3 to 16,113.3)	20.0 (13.5 to 30.0)	20,403.5 (13,228.8 to 32,163)	15.7 (10.1 to 24.6)	-0.99 (-1.09 to -0.89)	-21.6
CKD due to hypertension	172,320 (140,185.9 to 212,269)	291.8 (238.5 to 357.6)	350,326.4 (292,552 to 424,720.8)	258.7 (217.5 to 311.0)	-0.47 (-1.03 to 0.09)	-11.3
CKD due to T2D	189,886.8 (153,518.6 to 232,361.7)	316.5 (256.2 to 386.5)	381,113.3 (305,472.9 to 463,907.3)	273.9 (218.7 to 331.6)	-0.60 (-1.16 to -0.05)	-13.5

Values are point estimates with 95% uncertainty intervals (UIs) from GBD 2023. Absolute numbers refer to adults aged 20 years or older; age-standardised rates are the standard all-age GBD rates per 100 000 population. Estimated annual percentage changes (EAPCs) are presented with 95% confidence intervals (CIs) derived from the fitted log-linear trend models; these CIs reflect uncertainty in the estimated temporal trends and do not incorporate uncertainty in the underlying GBD estimates. AF/AFL=atrial fibrillation and flutter. CI=confidence interval. CKD=chronic kidney disease. CKM=cardiovascular–kidney–metabolic. DALY=disability-adjusted life-year. EAPC=estimated annual percentage change. GBD=Global Burden of Diseases, Injuries, and Risk Factors Study. HHD=hypertensive heart disease. IHD=ischaemic heart disease. MASLD=metabolic dysfunction-associated steatotic liver disease. PAD=peripheral artery disease. T2D=type 2 diabetes. UI=uncertainty interval.

Table S7. Projected CKM-related burden in Mexico, selected years, 2024–50.

Outcome	Metric	2024	2030	2040	2050
DALYs	Absolute number	8,997,435 (8,876,782–9,116,905; 8,813,890–9,181,564)	10,982,236 (10,595,466–11,379,616; 10,402,697–11,600,508)	15,330,754 (14,510,857–16,190,041; 14,122,752–16,700,411)	21,401,415 (19,952,725–22,891,492; 19,274,581–23,767,848)
DALYs	Age-standardised rate per 100,000	6,304 (6,194–6,411; 6,136–6,467)	6,323 (6,038–6,607; 5,888–6,749)	6,344 (6,053–6,635; 5,890–6,784)	6,347 (6,051–6,642; 5,902–6,800)
YLLs	Absolute number	6,922,393 (6,801,839–7,041,942; 6,736,795–7,108,150)	8,449,935 (8,062,680–8,855,278; 7,873,504–9,065,615)	11,760,120 (10,937,622–12,620,399; 10,531,164–13,115,532)	16,366,697 (14,896,663–17,873,980; 14,238,506–18,767,522)
YLLs	Age-standardised rate per 100,000	4,871 (4,761–4,981; 4,704–5,040)	4,916 (4,613–5,215; 4,450–5,381)	4,954 (4,625–5,269; 4,464–5,439)	4,961 (4,636–5,283; 4,463–5,447)
YLDs	Absolute number	2,075,042 (2,065,054–2,085,123; 2,059,680–2,090,529)	2,532,302 (2,447,491–2,620,091; 2,402,973–2,668,678)	3,570,635 (3,358,797–3,796,332; 3,247,117–3,913,090)	5,034,718 (4,644,936–5,444,837; 4,456,230–5,666,094)
YLDs	Age-standardised rate per 100,000	1,434 (1,430–1,439; 1,428–1,441)	1,457 (1,438–1,477; 1,427–1,487)	1,489 (1,459–1,520; 1,444–1,535)	1,521 (1,483–1,560; 1,463–1,579)

DALY=disability-adjusted life-year. YLL=year of life lost. YLD=year lived with disability. Each cell is reported as point estimate (80% prediction interval; 95% prediction interval). Absolute numbers refer to adults aged 20 years or older; age-standardised rates are per 100,000 population. Absolute DALY, YLL, and YLD trajectories were reconciled using MinT-shrink; rate series were modelled independently. Prediction intervals were derived from 10,000 simulated trajectories and represent forecasting uncertainty, not GBD uncertainty intervals.
